# Anomalous Origin of Right Coronary Artery from Distal Left Circumflex Artery: A Case Study and a Review of its Clinical Significance

**DOI:** 10.5681/jcvtr.2014.027

**Published:** 2014-06-30

**Authors:** Leili Pourafkari, Mohammadreza Taban, Samad Ghaffari

**Affiliations:** Cardiovascular Research Center, Tabriz University of Medical Sciences, Tabriz, Iran

**Keywords:** Coronary Anomaly, Myocardial Infarction, Coronary Angiography

## Abstract

Single coronary arteries are rare congenital anomalies in which the whole heart circulation is supplied by a coronary artery arising from a single ostium. Single left coronary artery with right coronary artery (RCA) originating from distal left circumflex artery (LCX) is a very rare anomaly with only few cases reported in the literature. We report a 44 years old male presenting with anterior myocardial infarction who was found to have a single left coronary artery during angiography. RCA had an abnormal origin arising from distal of a dominant LCX that retrogradely followed the course of a normal RCA to the base of the heart. A brief review of the reported cases with emphasis on the clinical significance of this unusual anomaly is presented.

## Case History

**Figure 1:**
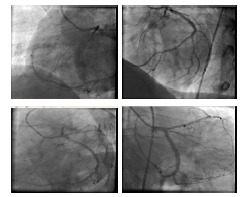
Retrograde filling of RCA from distal LCX shown in LAO (A), LAO cranial (B), LAO caudal (C) and shallow RAO with deep caudal (D) projections . A-C are before and D is after PCI.

A 44 years old man was referred to our hospital for coronary angiography. He had a history of anterior myocardial infarction four days earlier for which he had received streptokinase in another hospital and had been referred to our center for coronary angiography for recurrent ischemic symptoms. His past medical history was otherwise unremarkable. He didn’t report a history of smoking. He had developed recurrent chest pain on the third day of his admission that had been refractory to intensification of anti-ischemic therapy. Transthoracic echocardiography showed a left ventricular ejection fraction of 45%, hypokinetic anterior and apical segments and trivial mitral regurgitation. Right ventricular (RV) size and function were normal. He was scheduled for coronary angiography. During catheterization only one coronary ostium originating from left coronary cusp could be cannulated and several attempts with different catheters to identify the right coronary artery (RCA) ostium failed. Injection of contrast medium didn’t show any coronary artery originating from right coronary cusp. The patient had a single coronary artery arising from left coronary cusp. RCA had an abnormal origin arising from distal of a dominant left circumflex artery (LCX) that retrogradly followed the course of a normal RCA to the base of the heart (Figure 1). Left anterior descending artery (LAD) was cut off just after first septal branch with no angiographically visible antegrade or retrograde distal flow. A bare metal stent was deployed. The patient’s symptoms resolved completely following the procedure and he was discharged 2 days after percutaneous coronary intervention (PCI) without any complication. A myocardial perfusion scan performed six months after the index event showed scar tissue in anterior myocardial wall. Other segments did not show any abnormality. The patient was asymptomatic in 3 years follow up.

## Discussion

Single left coronary artery with anomalous origin of right coronary artery arising as a continuation of distal left circumflex artery is a very rare congenital coronary anomaly with few reported cases in the literature.^1-22^ Table 1  summarizes the demographics, angiography data, associated conditions, treatment options and follow-up data for the reported cases. Nine female and 15 male patients (age range: 30-77 years) have been reported (Table 1 ).^1-22^ This anomaly is compatible with L1 type of extensively used Lipton classification of coronary anomalies in which a single coronary artery from left sinus of valsalva divides to LAD and LCX, and distal LCX continues its course beyond the crux in to the atrioventricular groove and follows the course of a normal RCA to the base of the heart.^6,12^ Right coronary ostium is congenitally absent. Though single coronary arteries are often associated with other congenital anomalies^12^ and could be associated with the development of cardiac ischemia, cardiomyopathy, sudden cardiac death and congestive heart failure^14^, this particular anomaly has been reported to have a clinically benign course unless there are significant atherosclerotic lesions compromising the coronary flow.^10,12,14,20^ Majority of reported cases had a benign course and negative ischemic work up in the absence of coronary lesions.^2,4,6,11-14,21^ Choi et al. report a similar patient who presented with atypical chest pain. They attributed her chest discomfort to possible myocardial ischemia from abnormally slow coronary flow to the RCA and successfully treated the patient with calcium channel blocker and nitrates.^13^ On the other hand a 30 years old male with chest discomfort had mild posterolateral ischemia on perfusion imaging in the absence of any atherosclerotic lesion.^4^ Association with atrial fibrillation (AF) and severe mitral regurgitation (MR) have also been reported.^7,18^ Ma et al. report a similar patient who presented with right ventricular infarction and was treated with coronary stenting in distal LCX.^19^ Incidental finding during coronary CT angiography for the evaluation of atypical chest pain has been described.^20^ Ghaffari et al. described a patient with prolonged hemodynamic instability following a massive pulmonary embolism who was found to have a single left coronary artery. They attributed the prolonged and disproportionate RV dysfunction to its insufficient perfusion in the setting of acute pulmonary hypertension and absence of proximal RCA.^15^

**Table 1:** Summary of characteristics of reported cases with this unusual anomaly

**Case**	**Author/Year**	**Age/Sex**	**Presenting Symptom**	**Angiography**	**Associated Conditions**	**Further imaging**	**Treatment**	**Outcome**
1	Tavernarakis 1986	57/M	TCP	LAD lesion	None	None	NA	NA
2	Sheth 1988	60/M	ATCP	No lesion	None	None	None	NA
3	Vrolix 1991	51/M	TCP	LCX lesion	None	None	CABG	
4	Shammas 2001	44/F	Chest pain	No lesion	None	None	None	NA
5	Shammas 2001	30/M	Dyspnea/chest discomfort	No lesion	None	Mild posterolateral ischemia in MPI	None	NA
6	Turhan 2003	52/M	ATCP	No lesion	None	None	None	NA
7	Asha 2003	62/M	UA	LCX & LAD lesion	None	None	CABG	Uneventful recovery
8	Yoshimoto 2004	63/M	ATCP	No lesion	Atrial fibrillation	None	Oral anticoagulation for AF	NA
9	Chou 2004	42/M	TCP	40% lesion in LCX	None	Anteroapical ischemia in MPI	Medical	Asymotimatic at 1.5 yrs f/u
10	Kunimasa 2007	61/M	MI	LAD lesion	None	MSCT	NA	NA
11	Celik 2008	57/M	TCP	No lesion	None	Normal MPI	Medical	Asymptomatic at 1 yr f/u
12	Tanawuttiwat 2009	44/F	ATCP	No lesion	None	Normal DSE	Medical	NA
13	Datta 2010	69/F	TCP	No lesion	None	None	None	Asymptomatic at 1 yr f/u
14	Choi 2010	68/F	ATCP	No lesion	None	Normal MPI	NA	Symptoms resolved with CCB and nitrate
15	Chung 2010	77/F	TCP	LAD lesion	None	Normal MPI	PCI on LAD	NA
16	Ghaffari 2010	65/F	Dyspnea	No lesion	Massive pulmonary embolism	None	Medical	Dyspnea at 3 months f/u
17	Voyce 2010	76/F	RVMI	LAD and LCX lesion	None	None	PCI on LCX	Asymotimatic at 3 yrs f/u
18	Sonmez 2011	63/F	Subacute MI	LAD lesion	None	None	PCI on LAD	NA
19	Turfan 2012	58/M	exertional dyspnea and chest pain	Mid LAD lesion	Severe mitral regurgitation	None	Mitral valve surgery	NA
20	Ma 2012	39/M	RV MI	Distal LCX occlusion	None	None	PCI on LCX	NA
21	Blaschke 2013	59/F	TCP	No lesion	None	Negative DSE and Stress-perfusion cardiac MRI	None	NA
22	De Augustin 2014	40/M	ATCP	No lesion	None	Inconclusive EST,MSCT	Conservative	NA
23	Pourbehi 2014	47/M	MI	LCX & LAD lesion	None	None	PCI	Asymptomatic at 8 months f/u
24	Present case	44/M	MI	LAD lesion	None	None	PCI	Asymptomatic at 3 years f/u

ATCP=atypical chest pain, TCP= typical chest pain, PCI= percutaneous coronary intervention, MI= myocardial infarction, M=male, F= female, DSE= dobutamine stress echocardiography, MPI= myocardial perfusion imaging, UA= unstable angina, AF=atrial fibrillation, CABG= coronary artery bypass grafting, f/u=follow-up, RV=right ventricle, CCB= calcium channel blocker, NA= not available

Our patient similar to most of the reported cases didn’t have objective evidence of ischemia in the territory of RCA. Anomalous origin of RCA from distal continuation of LCX though extremely rare, seems to be an isolated and benign congenital anomaly in the absence of atherosclerotic lesions and it is unlikely that the anomaly causes myocardial ischemia. Actually left ventricular perfusion in these patients is very similar to that of normal subjects with LCX dominant coronary system. The main difference could be RV perfusion through RV branches. We postulated that the most vulnerable segments to ischemia in these patients could be in RV as described in few case reports of acute RV strain in the setting of pulmonary embolism^15^ or RV infarction since collateral circulation from proximal to distal RCA are not developed.^16,19^ Associated conditions are extremely uncommon and only one case of AF and one patient with severe MR are described in the literature. However coronary lesions could be of more critical significance because of the dependence of the heart’s circulation on a single coronary. Coronary artery bypass grafting and PCI have been described in a few cases with associated coronary atherosclerosis.

## Ethical issues

The study was approved by the Ethics Committee of the University.

## Competing interests

Authors declare no conflict of interest in this study.
